# Rock-making bacteria: manganese oxidation and biomineralization in pseudomonads

**DOI:** 10.1128/jb.00377-26

**Published:** 2026-09-18

**Authors:** Kati Geszvain, Hope Johnson, Jasquelin Peña, Bradley M. Tebo

**Affiliations:** 1 Department of Biological Sciences, California State University Chico 585532, Chico, California, USA; 2 Department of Biological Sciences, California State University Fullerton 585532, Fullerton, California, USA; 3 Department of Civil and Environmental Engineering, University of California 166630, Davis, California, USA; 4 Earth and Environmental Sciences Area, Lawrence Berkeley National Laboratory 1666 , Berkeley, California, USA; 5 Scripps Institution of Oceanography, University of California 8784, San Diego, California, USA; Dartmouth College Geisel School of Medicine, Hanover, New Hampshire, USA

**Keywords:** pseudomonads, *Pseudomonas putida *GB-1, manganese (Mn) oxidation, multicopper oxidases, MnxG, heme peroxidases, biofilm, siderophores, phenotypic heterogeneity, biomineralization

## Abstract

The oxidation of manganese from Mn(II) to Mn(III) or Mn(IV) is a common trait in the *Pseudomonas* genus. Many pseudomonads oxidize Mn enzymatically using one or more multicopper oxidases (MnxG and McoA) and in some cases a heme peroxidase (MopA). The oxidation of Mn leads to the extracellular precipitation of reactive Mn oxides in association with extracellular polymeric substances (EPS). Here, we review the current state of knowledge on Mn oxidation and biomineralization in pseudomonads. While most of the genetic, biochemical, and physiological insights we present derive from studies on *Pseudomonas putida* GB-1, we also examine the mechanisms of Mn oxidation in other Mn-oxidizing pseudomonads and survey the potential for Mn oxidation across the genus through phylogenetic analysis. Although the physiological and microbial community function(s) for Mn oxidation and biomineralization have yet to be elucidated, we discuss possible roles for Mn oxidation and identify key knowledge gaps regarding the mechanism and function of this trait in the *Pseudomonas* genus.

## INTRODUCTION

**Fig 1:**
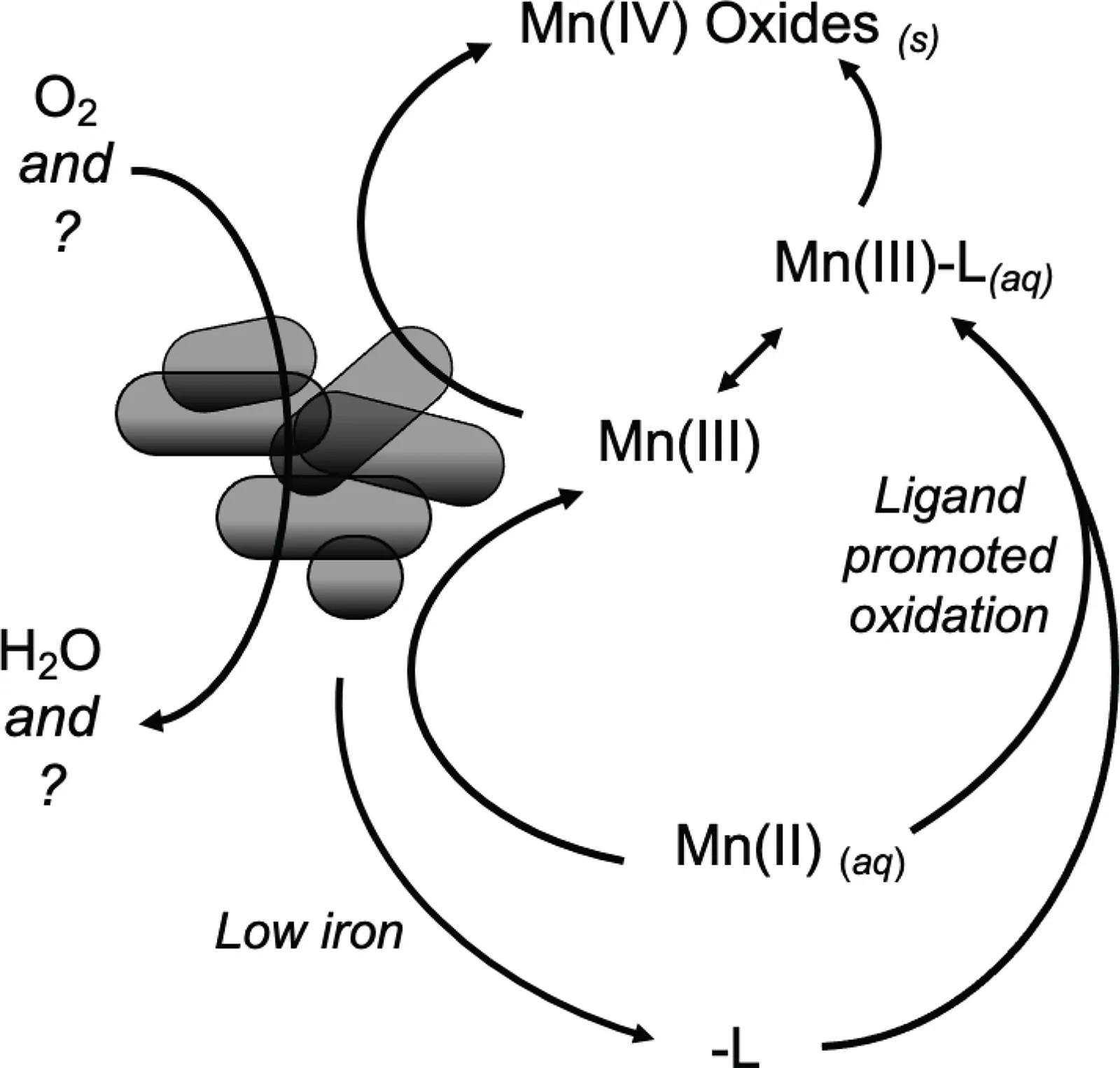
The oxidative manganese cycle in *Pseudomonas*. Enzymatic oxidation of Mn(II) to Mn(IV) oxides proceeds via two one-electron transfer reactions with Mn(III) as an intermediate. Mn(III) may be trapped extracellularly as Mn(III) complexes in the presence of complexing inorganic or organic ligands (Mn(III)–L), which may remain in solution, disproportionate to Mn(II) + Mn(IV) oxides, or be further oxidized to Mn(IV) oxides. Under iron-limiting conditions, bacteria may produce siderophores that promote the abiotic oxidation of Mn(II) to Mn(III)–L. (After Tebo et al. (6), with permission from Springer).

The ability of microorganisms to precipitate minerals has deep implications for Earth’s history, the evolution and metabolism of myriad organisms, and the biogeochemical cycling of carbon, nutrients, and metals (1–4). For the transition metal manganese (Mn), biomineralization is a critical component of its biogeochemical cycle, as biological activity catalyzes the oxidation of Mn(II) to form Mn(III) and biogenic Mn(IV) oxides (Fig. 1). This process can be carried out by a diverse group of organisms that oxidize Mn either indirectly through the production of metabolites like reactive oxygen species (ROS) or enzymatically through the use of multicopper oxidases (MCOs) and/or heme peroxidases (5).

This minireview focuses on *Pseudomonas* bacteria that are known or have the genetic potential to oxidize manganese (Mn) enzymatically. Manganese oxidation is widespread among *Pseudomonas* species and has been studied most in *P. putida* strains GB-1 and MnB1, with some investigation in *P. putida* KT2440, *Pseudomonas* sp. strains MS-1 and DSV-1, and *P. resinovorans* species. Other Mn-oxidizing pseudomonads have been isolated from a variety of environments, including marine oxic and anoxic interfaces (7), mid-ocean ridges (8), caves (9), groundwater (10), lake sediments (11), compost (12), soil (13–15), wetlands (16), and potable water systems (17), to name a few.

In the sections that follow, we begin with a primer on the significance of Mn in the environment. We then present the current state of knowledge on the genetics, biochemistry, and physiology of known Mn-oxidizing *Pseudomonas* species. In the final section, we explore potential functions for Mn oxidation and highlight open questions in this field. While bacterial Mn oxidation has been recognized for over a century (18, 19), its biochemical mechanisms, regulatory pathways, and response to environmental conditions are only partially understood, and its physiological and/or community function(s) are largely unknown. Research on Mn oxidation and biomineralization in pseudomonads is poised to answer many of these questions, aided not only by existing foundational literature but by the three key properties of pseudomonads: they are ubiquitous, can be cultured with ease, and are amenable to genetic manipulation.

## ENVIRONMENTAL SIGNIFICANCE AND CHEMISTRY OF MANGANESE

Manganese is the second most abundant transition metal in the Earth’s crust (20). Its biological functions include oxygen production in oxygenic photosynthesis, protection against superoxide, DNA repair and replication, among others (21). In nature, Mn can cycle between three oxidation states, II, III, and IV (22). Mn(II), the form that is most biologically available to organisms, occurs as an aqueous ion (Mn(H_2_O)_6_^2+^) in solution, forming weak metal complexes with a variety of anion ligands, or mineral phases such as Mn carbonate, Mn phosphate, and Mn sulfide. Mn oxidation, which can occur through both chemical and biological mechanisms, is thermodynamically favorable but kinetically slow under common environmental conditions (22). Biological catalysis, however, allows for much faster reaction kinetics, producing both Mn(III) and Mn(IV) (Fig. 1) and providing the primary pathway for Mn(IV) oxide precipitation (5, 23).

Mn(IV) is highly insoluble in water and tends to undergo redox reactions. It occurs in the form of Mn oxides, typically as layer-type minerals that can be found as discrete particles (e.g., in the water column), as coatings on surfaces (e.g., in soil and sediment), and in association with Fe oxides forming ferromanganese deposits (20). In the absence of oxygen, Mn(IV) oxides can serve as an electron acceptor for anaerobic bacterial respiration (24, 25). They are among the strongest solid-phase oxidants in natural systems (26, 27) and have high sorption reactivity toward other transition metals, oxyanions, rare earth elements, and radionuclides (2, 28–30). Given this high reactivity, Mn oxides impact many biogeochemical cycles, environmental processes, and technological applications (2, 5).

The intermediate redox state, Mn(III), forms both soluble and insoluble species but is distinguished by its instability as a free aqueous ion (Mn^3+^) (22). Mn(III) can form Mn(III) oxide minerals or be incorporated into or adsorbed by Mn(IV) oxides (20). As an aqueous ion, Mn^3+^ will rapidly disproportionate to Mn(II) and Mn(IV) unless it is stabilized by a complexing ligand such as pyrophosphate, oxalate, or organic biomolecules such as siderophores, which are produced by bacteria, fungi, and roots primarily for iron acquisition (31–34). Ligand-bound Mn(III) is much more abundant and prevalent in the environment than previously thought, with marine/estuarine concentrations of up to 7 µM and 86% of the total dissolved Mn (35–37). In organic-rich soils, fungi produce organic Mn(III) complexes, which participate in the degradation of organic matter (30). Finally, Mn(III) can serve as both an electron acceptor and an electron donor and therefore is a reactive intermediate that affects the redox chemistry of many other chemical species and plays an important role in carbon, sulfur, and metal cycles in the environment (38).

## GENETICS, BIOCHEMISTRY AND PHYSIOLOGY OF KNOWN MN-OXIDIZING *PSEUDOMONADS*

### *Pseudomonas putida* GB-1

**Fig 2:**
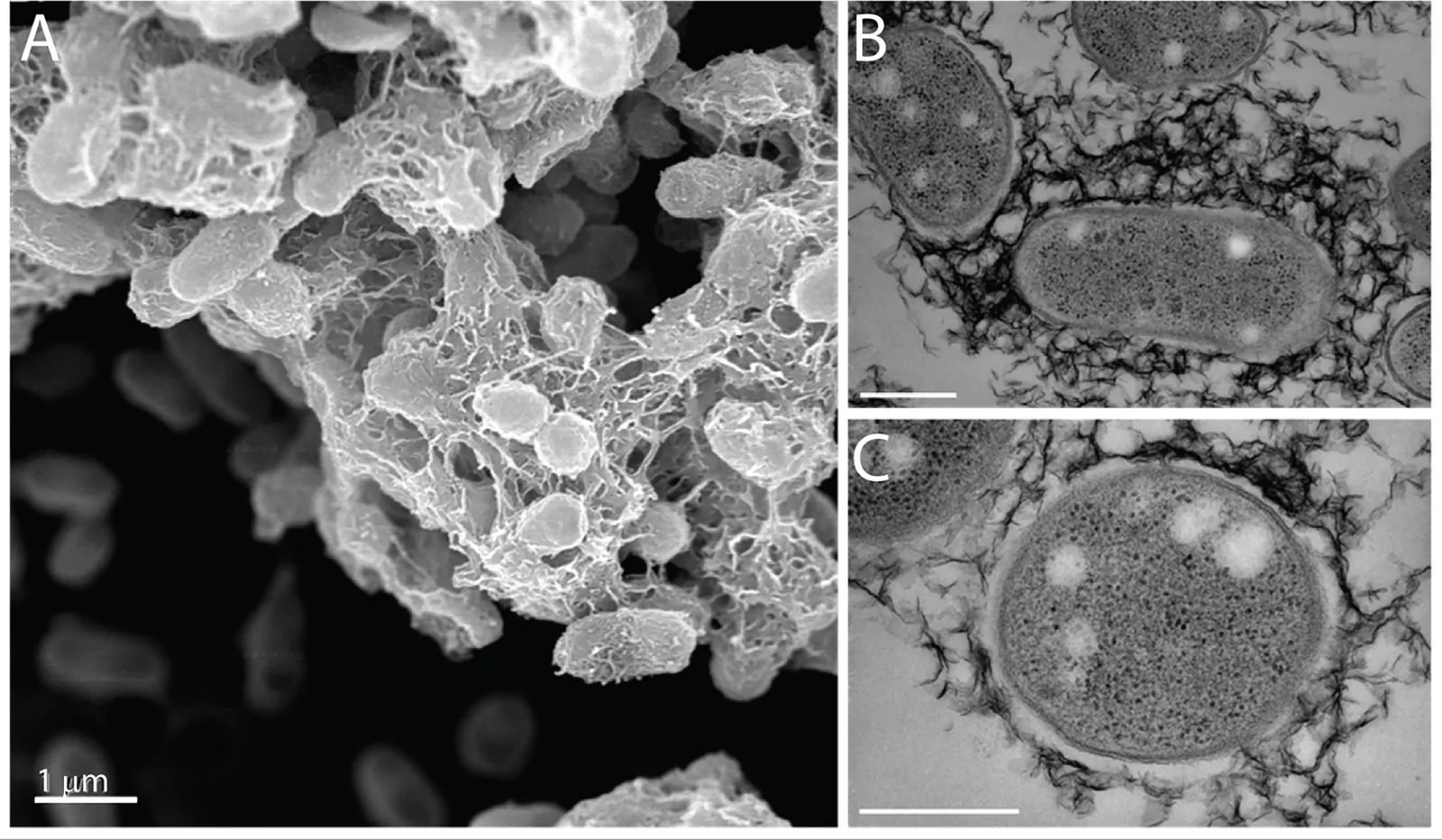
Electron micrographs of *Pseudomonas putida* GB-1 propagated in liquid culture using LEPT medium supplemented with 1 mM MnCl_2_. Scanning electron micrograph (**A**, 1-micron scale bar) shows suspended cells enmeshed in an EPS matrix, while some planktonic cells free of EPS can be observed in the background. Transmission electron micrographs (**B** and **C**, 0.5-micron scale bar) illustrate the extracellular distribution of biogenic Mn oxide, which appears as agglomerated electron-dense particles with sheet-like morphology that extend outward from the cell membrane but are rarely in direct contact with the cell surface. For SEM, the sample was fixed in 2% glutaraldehyde overnight, rinsed four times in sodium cacodylate buffer, and postfixed with osmium tetroxide prior to a 7-step ethanol dehydration sequence. Samples were dried in a critical point dryer, mounted, and coated with 10 nm carbon. Images were collected using a Hitachi 5000 microscope. For TEM, samples were prepared by high-pressure freeze (LEICA EMPACT) and freeze substitution using 2% osmium tetroxide and 0.1% uranyl acetate in acetone. After a 72 h thawing cycle, samples were washed, infiltrated with resin, embedded at 60°C for 72 h, and finally thin-sectioned to 60 nm using a diamond knife. Images were collected using a Tecnai 12 microscope.

While the ability to oxidize Mn(II) is found in diverse bacteria (5), *Pseudomonas putida* GB-1 (ARS Culture Collection (NRRL) accession number B-65545; Leibniz-Institut DSMZ-Deutsche Sammlung von Mikroorganismen und Zellkulturen GmbH number DSM 120,756) has been used as a model Mn-oxidizing bacterium for almost 30 years (39) due to its sequenced genome (accession #NC_010322.1) and genetic tractability. The strain was isolated from Mn micronodules in sediment from Lake Michigan in Green Bay, Wisconsin (Kenneth Nealson, personal communication) and initially named *Pseudomonas fluorescens* GB-1 (39). Later, the strain was renamed *P. putida* GB-1 based on its 16S rRNA gene sequence (40). In laboratory cultures, Mn oxidation results in a visible brown precipitate, with Mn oxides accumulating extracellularly as illustrated in Fig. 2 (41). The brown precipitate phenotype is regularly used as a proxy for Mn(II) oxidation, although Mn(II) may be oxidized only to Mn(III) or environmental and cellular conditions may lead to concurrent or subsequent reduction of the Mn(IV) to Mn(III) or even Mn(II) (5). In *P. putida* GB-1, Mn oxides appear at the beginning of the stationary phase with a maximum oxidation rate at neutral pH and 26% oxygen saturation (39) and form nanoscale layer-type Mn(IV) oxides (42, 43). Manganese oxidation can occur at concentrations of up to and greater than 1 mM in complex media (44). Additionally, Mn oxidation can be stimulated by the addition of Cu^2+^ but not Zn^2+^ or Ni^2+^ (45, 46).

### The enzymes responsible for Mn oxidation

**Fig 3:**
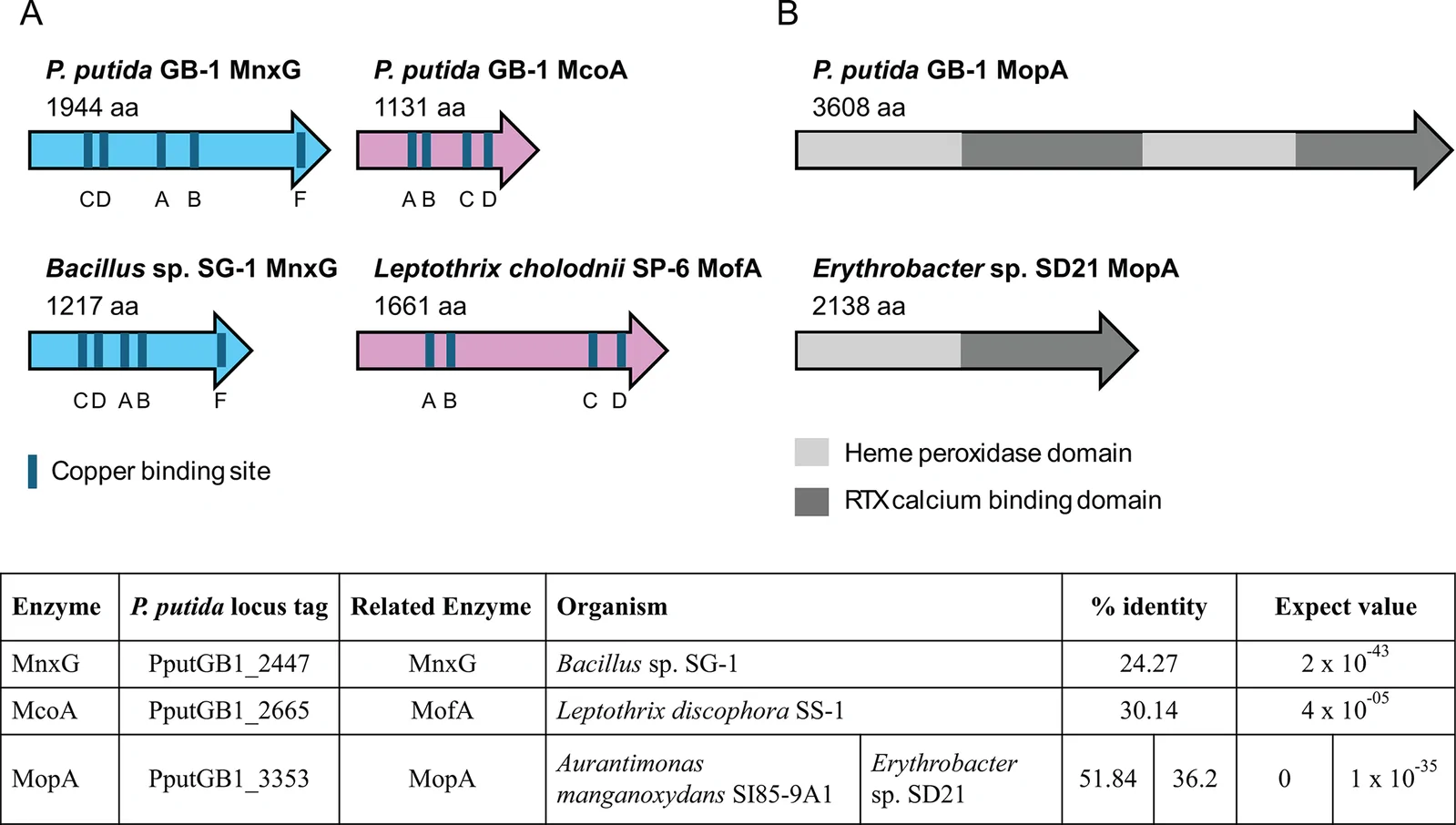
Mn-oxidizing proteins in *P. putida* GB-1. (**A**) Size and Cu-binding site distribution of Mn(II)-oxidizing MCO genes. The Cu-binding motifs of MnxG and McoA are shown in blue. The letters indicate the arrangement of the different copper-binding regions. (**B**) MopA Mn-oxidizing proteins contain a heme peroxidase domain and a calcium-binding domain with several calcium-binding sites. The *P. putida* GB-1 MopA has repeated domains, similar to *Aurantimonas* sp. SI-9A1.

**Fig 4:**
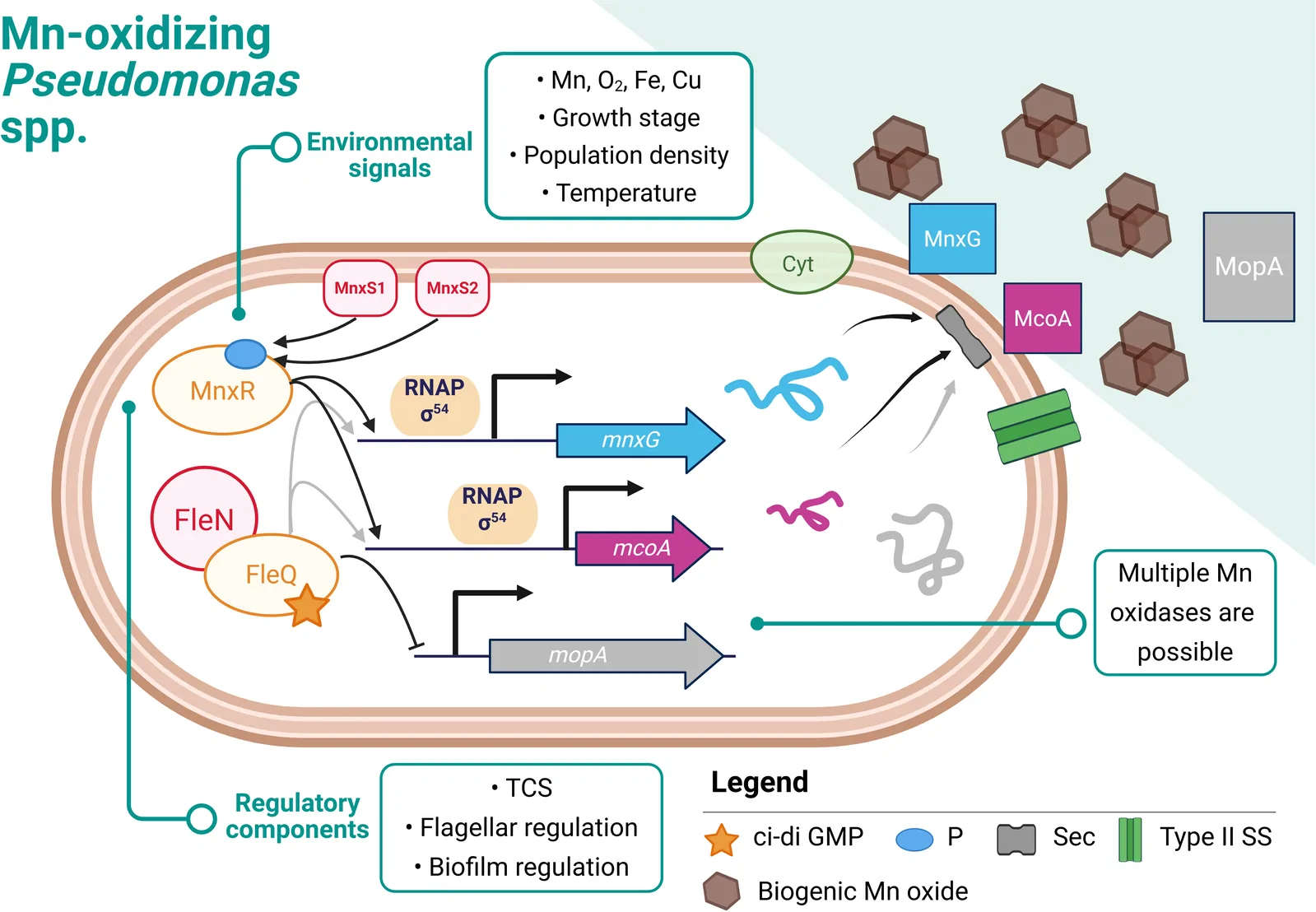
Proposed model of Mn oxidation in *P. putida*, including known regulatory components. In the TCR pathway, sensor kinases MnxS1 and MnxS2 phosphorylate the response regulator MnxR. Phosphorylated MnxR is shown positively regulating both *mnxG* and *mcoA*; it is not known if MnxR also regulates *mopA* expression. FleQ bound to the second messenger c-di-GMP positively regulates *mnxG* and *mcoA* and represses *mopA* expression, while FleN modulates FleQ activity. Growth stage, population size, the concentration of Mn(II), oxygen, and nutrients are all possible environmental signals, resulting in regulated expression of the oxidase genes. MnxG and McoA are transported through the Sec general secretion system to the periplasm. One or more of the three oxidases are transported across the outer membrane by a novel type II secretion system. Mn oxidase activity and Mn oxides are found on the outside surface of the bacterium. Oxidation of Mn(II) may be mediated by the presence of cytochromes in the membrane.

The *P. putida* GB-1 genome encodes three Mn oxidase enzymes—MnxG, McoA, and MopA (Fig. 3) (47, 48). These three enzymes are briefly described below and highlighted in Fig. 4. We note that CumA was previously identified as a Mn-oxidizing enzyme (45), but this was shown to be incorrect (49). MnxG (1,944 amino acids) and McoA (1,131 amino acids) are multicopper oxidases (MCOs; Fig. 3), which employ multiple copper centers to transfer electrons one by one (50) from the substrate (Mn) to molecular oxygen. Both MnxG and McoA can oxidize Mn(II) to Mn(III) and Mn(III) to Mn(IV), resulting in a Mn(IV) oxide product. *P. putida* GB-1 mutants expressing either MnxG or McoA are able to produce Mn(IV) oxides, but a nonoxidizing phenotype can be achieved by deletion of both *mnxG* and *mcoA* genes (48, 51). MnxG and McoA have five and four, respectively, conserved putative copper-binding domains (Fig. 3). The arrangement of these Cu-binding domains also differs between the two proteins, suggesting they did not arise by gene duplication (6, 52). While the biochemical characteristics of MnxG and McoA from *P. putida* GB-1 have not been investigated, MnxG from the Mn-oxidizing *Bacillus* sp. PL-12 (Fig. 3) has been fully characterized (53) and serves as a model for how MnxG from *P. putida* and other Mn-oxidizing MCOs, such as McoA, may function.

In Mn(II)-oxidizing strains of various marine *Bacillus* species, MnxG localizes to the exosporium (54, 55) in a complex with a hexamer of two small accessory proteins (MnxE and MnxF) (56, 57). Cryo-EM and mass spectrometric analysis revealed that the structure of the Mnx complex is composed of MnxG capped by a hexameric ring of alternating MnxE and MnxF subunits. A tunnel runs through the entire pear-shaped complex with Mn^2+^ binding at the entrance. Sequential one-electron oxidation reactions occur along the tunnel, with Mn(IV) ultimately condensing into MnO_2_ nanoparticles as the Mn(IV) exits the protein through the MnxE_3_F_3_ ring (58). The structure of the *Bacillus* Mnx complex raises the question of whether accessory proteins are needed for activity of the MCOs in pseudomonads or whether MnxG and McoA are colocalized and work cooperatively. The latter is suggested by Gehin et al. (59) based on enhanced kinetics of Mn oxidation in the wild-type strain relative to the single-deletions strains (see Environmental conditions influence *mnxG* and *mcoA* gene expression); however, this needs further investigation.

The MopA protein (3,608 amino acids) of *P. putida* GB-1 is similar to the MopA found in the Mn(II)-oxidizing alphaproteobacteria *Aurantimonas manganoxydans* SI85-9A1 and *Erythrobacter* sp. SD-21 (Fig. 3) (60). The MopA-like Mn oxidases are characterized by having one or more animal heme peroxidase domains upstream of a calcium-binding domain (Fig. 3), although there is no evidence that the MopA enzymes use hydrogen peroxide for Mn oxidation (61, 62). MopA enzymes oxidize Mn(II) to Mn(III), which then likely disproportionates extracellularly to form Mn oxides (63). In *P. putida* GB-1, MopA is secreted (47). Its contribution to Mn oxidation in *P. putida* GB-1 was undetectable in the wild type (48) and the *mnxG* and *mcoA* double knockout (51). However, Mn oxidation was observed in a strain from which *mnxG*, *mcoA,* and a regulatory gene (*fleQ*, see below) were all deleted (47). Manganese oxides in this strain appeared later, at a lower concentration relative to wild type, and were located away from the the colony on solid media. These features suggests the enzymatic oxidation of Mn(II) to Mn(III) by MopA with subsequent disproportionation to Mn(IV) (47). The environmental or growth conditions under which MopA contributes to Mn oxidation in *P. putida* GB-1 remain to be elucidated.

### Transport and localization of Mn oxidases

**Fig 5:**
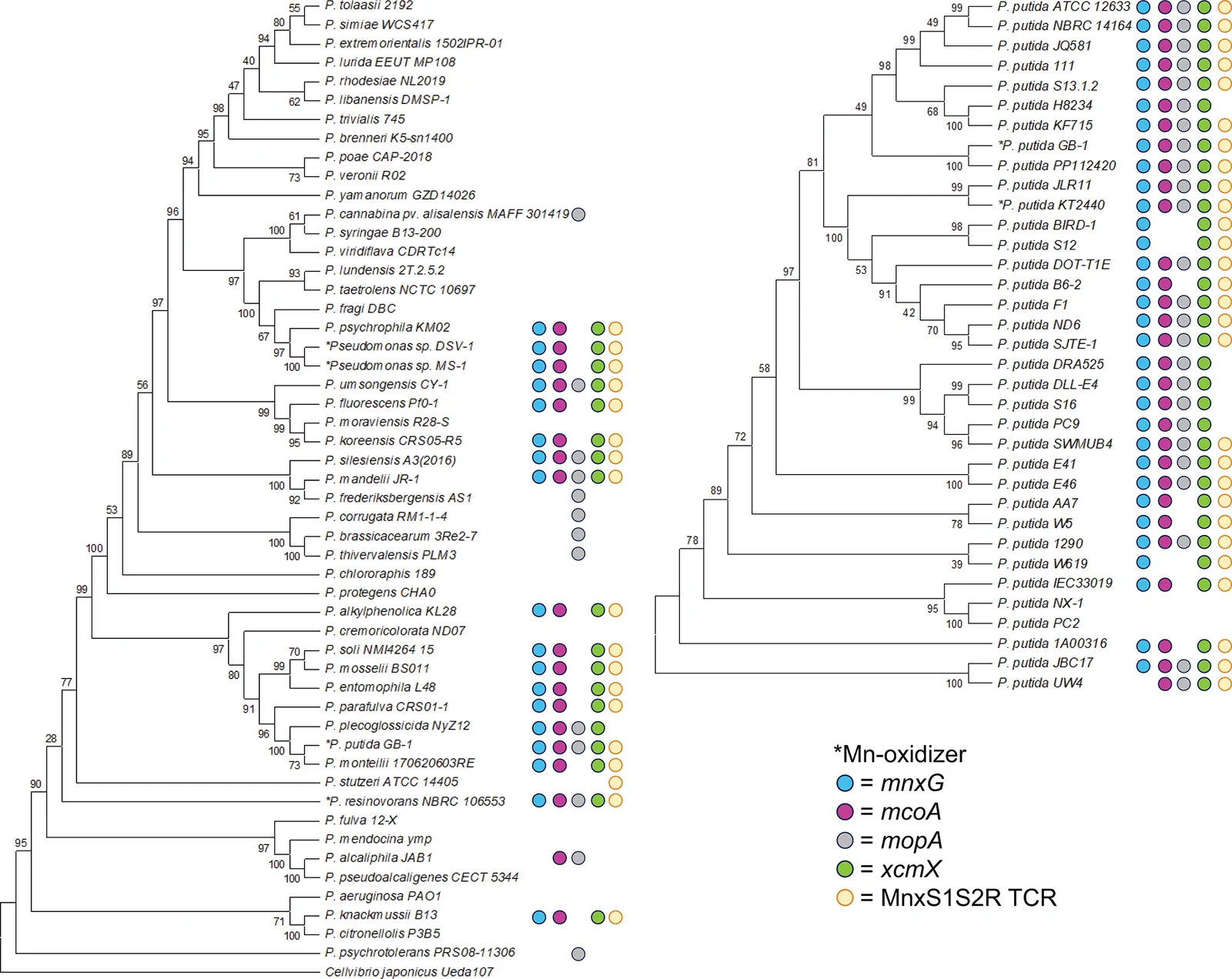
Survey of potential Mn-oxidizing bacteria in the *Pseudomonas* genus. Phylogenetic tree of potential Mn-oxidizing pseudomonads among species with complete genomes and phylogenetic tree showing potential Mn-oxidizing *P. putida* species. * denotes strains known or belonging to the same species as those known to oxidize Mn. BLAST was used to find homologs to *P. putida* GB-1 Mn oxidation genes *mnxG*, *mcoA*, *mopA,* and *xcmX*, using an E value cutoff of less than 1 x 10^−40^. To identify putative MnxS1S2R TCR operons, a cassette search was performed on the Integrated Microbial Genomes & Microbiomes system (66) for operons composed of two sensor kinases and one response regulator. The phylogeny was generated using the housekeeping gene *rpoD* and inferred using the maximum likelihood method and Tamura-Nei model (67) of nucleotide substitutions, and the tree with the highest log likelihood (−26,329.83) is shown. The percentage of replicate trees in which the associated taxa clustered together (1,000 replicates) is shown next to the branches (68). The initial tree for the heuristic search was selected by choosing the tree with the superior log-likelihood between a Neighbor-Joining (NJ) (69) and a maximum parsimony (MP) tree. The NJ tree was generated using a matrix of pairwise distances computed using the Tamura-Nei model (67). The MP tree had the shortest length among 10 MP tree searches, each performed with a randomly generated starting tree. The analytical procedure encompassed 52 *rpoD* DNA sequences or 34 *rpoD* DNA sequences using 1st, 2nd, 3rd, and noncoding positions with 2,032 positions in the final data set. Evolutionary analyses were conducted in MEGA utilizing up to four parallel computing threads (70).

The accumulation of Mn oxides on the cell surface (Fig. 2) suggests that Mn oxidation occurs outside the cell, necessitating the transport of the oxidases across the cell envelope. Both MnxG and McoA possess amino-terminal Sec system signal peptides, indicating that they are transported across the cell membrane by the Sec system (Fig. 4) (48). MopA lacks a signal peptide but can be detected in culture supernatant, suggesting it is also secreted (47). Further evidence for the export of these proteins is provided by work by the de Vrind group, which identified several transposon insertion mutants where intact cells failed to oxidize Mn, while cell lysates showed wild-type levels of Mn oxidation (64, 65). The transposon insertions mapped to genes with high similarity to type II secretion system (T2SS) genes, including *xcmX* (PputGB1_2462), a gene conserved in Mn-oxidizing pseudomonads (Table 1, Fig. 5A and B). This pathway may be specialized for the transport of Mn oxidases, as there is a second T2SS operon in the GB-1 genome with greater similarity to the *P. aeruginosa* T2SS.

**TABLE 1:** Potential Mn-oxidation-related genes in pseudomonads (% amino acid identity to GB-1 protein)

Gene	*P. putida*		Cold-tolerant *Pseudomonas*		*P. resinovorans*	
	GB-1^*a*^	KT2440^*a*^	DSV-1^*b*^	MS−1^*b*^	MO-1^*b*^	MOB 513^*a*^
*mnxR*	PputGB1_2521	PP3419 (97%)	2,986,952,694 (77%)	2,986,957,691 (77%)	2,808,397,378 (71%) 2,808,397,339 (69%)	UK
*mnxS1*	PputGB1_2519	PP_3421 (94%)	2,986,952,698 (61%)	2,986,957,687 (61%)	2,808,397,377 (55%) 2,808,397,338 (49%)	UK
*mnxS2*	PputGB1_2520	PP_3420 (95%)	2,986,952,695 (62%)	2,986,957,690 (62%)	2,808,397,377 (59%) 2,808,397,338 (51%)	UK
*mcoA*	PputGB1_2665	PP3184 (92%)	2,986,952,697 (74%)	2,986,957,688 (74%)	2,808,396,995 (74%)	Pres513_3296 (31%)
*mnxG*	PputGB1_2447	PP3490 (97%)	2,986,952,721 (79%)	2,986,957,664 (79%)	2,808,397,348 (74%)	Pres513_3924 (74%)
*xcmX*	PputGB1_2462	PP3475 (93%)	2,986,952,706 (62%)	2,986,957,679 (61%)	2,808,397,362 (60%)	UK
*mopA*	PputGB1_3353	PP2561 (95%)	NP	NP	2,808,396,093 (66%), 2,808,395,205 (56%), 2,808,394,831 (48%)	Pres513_7013 (55%), 7014 (52%), and 5806 (65%)
SCO1-1	PputGB1_2449	PP_3488 (92%)	2,986,952,719 (60%)	2,986,957,666 (60%)	2,808,397,350 (54%)	UK
SCO1-2	PputGB1_2450	PP_3487 (95%)	2,986,952,718 (64%)	2,986,957,667 (64%)	2,808,397,351 (69%)	UK
SCO1-3	PputGB1_2666	PP_3183 (94%)	2,986,952,696 (60%)	2,986,957,689 (59%)	2,808,396,996 (64%)	UK

^*a*^ IMG locus tag.

^*b*^ IMG gene number; UK, unknown; NP, not present.

### Regulation of Mn oxidation

**Fig 6:**
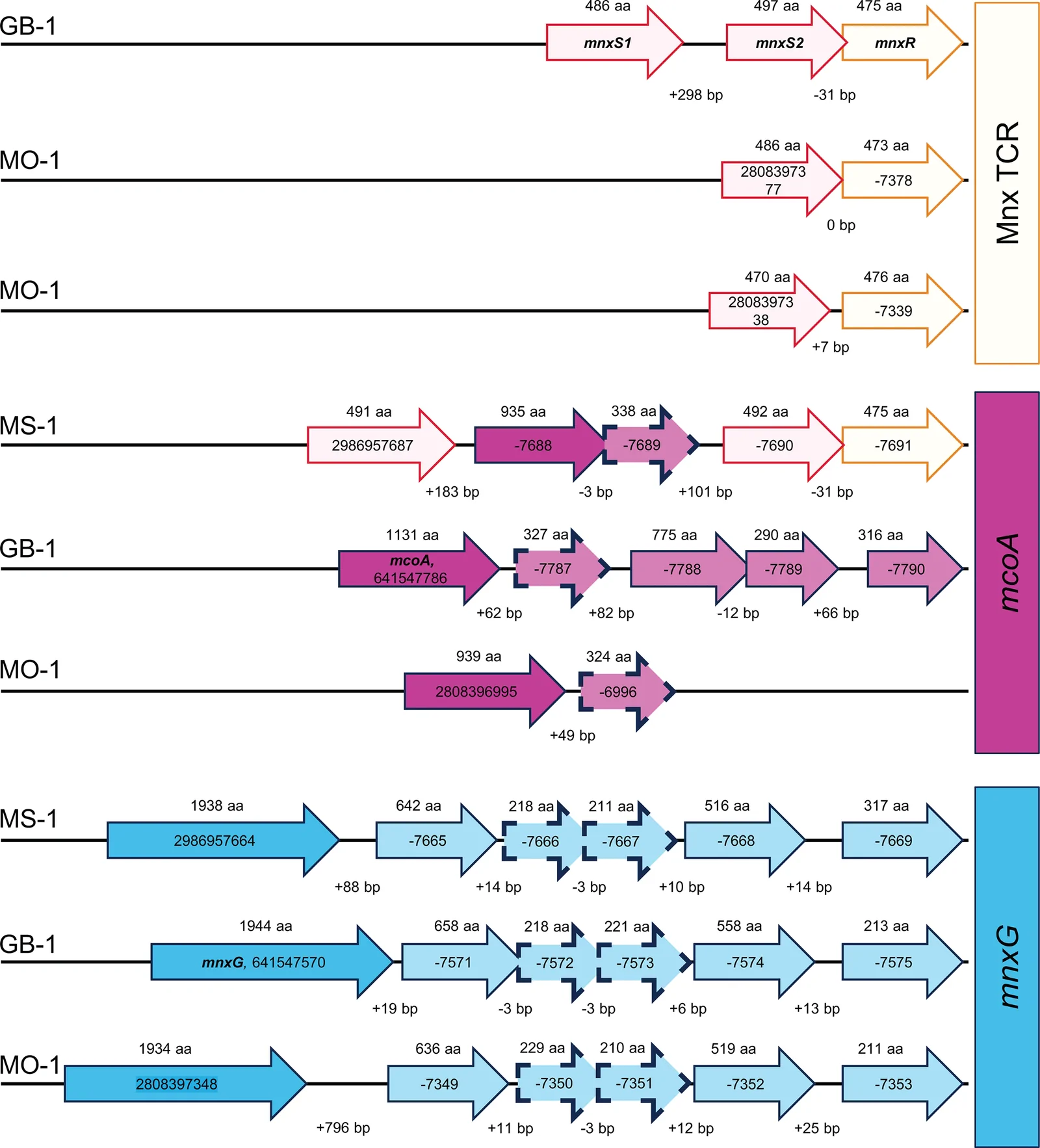
Putative Mn oxidation operons in known Mn-oxidizing pseudomonads. Arrows represent predicted genes. Numbers below the arrows represent the number of base pairs (bp) between predicted genes; numbers above the arrows are the length of the predicted protein product in units of amino acids (aa). Written within the arrows are the gene names and/or IMG Gene ID#. Genes in the putative *mnxG* operon are blue; genes in the *mcoA* operon are purple, and those in the MnxS1S2R two-component regulatory pathway are red and yellow. Putative SCO1/SenC genes are outlined in a black dashed line. GB-1: *Pseudomonas putida* GB-1; MS-1: *Pseudomonas* sp. MS-1 (DSV-1 not shown for simplicity; it is very similar to MS-1); MO-1: *Pseudomonas resinovorans* MO-1. *P. resinovorans* has two possible orthologs of MnxS1/2 and MnxR.

In *P. putida* GB-1, the Mn oxidation phenotype requires a two-component regulatory (TCR) pathway called the MnxS1S2R TCR (49) (Table 1, Fig. 4). In a TCR pathway, the first component is a sensor kinase (SK) that detects an environmental signal and subsequently undergoes autophosphorylation. This phosphate is then transferred to the second component, the response regulator (RR), which produces a response to the signal, usually through transcriptional regulation (71). As shown in Fig. 6, MnxS1S2R TCR consists of two SKs (*mnxS1* and *mnxS2*) and one RR (*mnxR*) located in a putative operon. While the reason for having two separate SKs, MnxS1 and MnxS2, is unknown, they are 59% identical and 72% similar to one another at the amino acid level. The MnxR RR is a putative σ^54^-dependent transcriptional activator (49), which is consistent with the occurrence of σ^54^-binding sites in the promoter regions of both *mnxG* and *mcoA* (51). In a number of different microorganisms, σ^54^ regulates genes involved in nitrogen response, flagella synthesis, motility, and biofilm formation (72, 73). Deletion of any of the MnxS1S2R genes results in a complete loss of the Mn oxidation phenotype (49). When *mnxR* is deleted, transcription of *mnxG* and *mcoA* decreases (note that *mnxS1* and *mnxS2* deletions were not evaluated and *mopA* was not screened) (48), suggesting that this TCR is likely involved in regulating transcription of at least two of the oxidase genes.

In addition to the involvement of a σ^54^ regulator, Mn oxidation may also be regulated by the integration host factor (IHF). The promoter regions of *mcoA* and *mnxG* have predicted binding sites for the integration host factor (IHF) (51), which is a global regulator of the transition into stationary phase (74). The presence of IHF binding sites in these promoters is consistent with the onset of Mn oxidation in stationary-phase growth.

In *Pseudomonas* spp., FleQ, another σ^54^-dependent transcription factor, controls the switch between planktonic and sessile lifestyles by regulating flagellar synthesis and biofilm formation (75–77). FleQ is regulated by the second messenger cyclic diguanylate monophosphate (c-di-GMP) and by FleN (75, 78). FleQ and FleN also regulate Mn oxidation in *P. putida* GB-1 (Fig. 4). Deletion of *fleN* restores Mn oxidation to the *mnxR* deletion strain, while deletion of *fleQ* from the *mnxR* deletion strain upregulates *mopA* (47). Because Mn oxides accumulate outside the cell in an EPS matrix (Fig. 2A) (44, 79), Mn oxidation is likely co-regulated with biofilm formation through the activities of FleN and FleQ.

Recent work shows that Mn oxidation is regulated at the population level (see below, (51, 59). Because Mn oxidation takes place outside the cell (Fig. 2) this activity may benefit other members of the community and could be dependent on cell-to-cell interactions, as shown with Mn-oxidizing consortia (80–82). Any community-level regulation of Mn oxidation is likely to involve quorum sensing, which in *P. aeruginosa* involves the Gac/Rsm system (83, 84). Disruption of *rsmA* by a transposon insertion resulted in increased Mn oxidation (85). RsmA is a regulatory protein that binds mRNAs, blocking their translation (83). Thus, Mn oxidation may also be regulated post-transcriptionally.

### Environmental conditions influence *mnxG* and *mcoA* gene expression

Through the use of reporter gene fusion strains, Gehin et al. (51) found that *mnxG* and *mcoA* are successively activated in stationary-phase cells irrespective of planktonic or sessile lifestyle. No gene expression or Mn oxide precipitation was observed in the absence of Mn(II) or in a Δ*mnxG*Δ*mcoA* double knockout strain, respectively, under any of the conditions tested (51, 59). The colocalization in microcolonies of Mn oxide formation and reporter expression in the *mnxG* but not *mcoA* reporter strains demonstrated that MnxG is responsible for the initial precipitation of Mn oxides, whereas McoA contributes to biomineralization under conditions where MnxG activity is saturated or inhibited. The observed patterns in promoter expression were consistent with the measured apparent whole-cell Mn(II) Km for MnxG of 26 µM and McoA of 117 µM (59). However, gene expression likely depends on environmental factors besides the Mn concentration. The comparison of gene expression patterns in microcolonies formed under oxygen-limiting versus oxygen-replete conditions suggested that low oxygen may decrease *mnxG* expression, while enhancing *mcoA* expression (51). The multiple Mn oxidases in *P. putida* GB-1 thus appear to facilitate Mn oxidation under a broad range of environmental conditions.

Time-lapse imaging of the reporter strains also revealed that Mn oxidation is differentially regulated among individuals within a population (59). Specifically, a concentration-dependent response in gene activation was observed, where the proportion of individuals activating *mnxG* increased with increasing Mn(II) concentration up to a threshold concentration of 10 µM Mn(II). Once the full population activated *mnxG*, then *mcoA* expression occurred, with *mcoA* expression also following a concentration-dependent behavior until most of the population activated *mcoA* (10–500 µM Mn(II)). Subsequent experiments using single-gene deletion strains identified MnxG as the dominant oxidase, with faster rates of Mn oxide precipitation for the MnxG-only strain than the McoA-only strain at low Mn concentrations. Wild-type rates were greater than the sum of the rates measured in the single-gene deletion strains at high Mn concentrations, which suggests that gene coexpression and/or interaction between MnxG and McoA may be required for full conversion of Mn(II) to Mn oxide at high Mn(II) concentrations. The regulation of *mnxG* and *mcoA* expression by Mn(II) concentration supports the conclusion that the function of MnxG and McoA is Mn oxidation, but the underlying mechanism by which these proteins work together to precipitate Mn oxides remains an open question.

### Siderophores promote Mn(III) formation and inhibit Mn oxide formation

Early investigations into the effect of Fe on Mn oxidation in *P. putida* MnB1 (discussed in “Mn oxidation in other pseudomonads” and “*P. putida* MnB1”) revealed that under Fe-limiting conditions, Mn oxide formation was very slow or did not occur (B. M. Tebo, unpublished). Subsequent work showed that under Fe-limiting conditions, fluorescent pseudomonads like *P. putida* GB-1 and MnB1 produce the fluorescent siderophore pyoverdine (PVD). This siderophore complexes Mn(III) and inhibits the formation of Mn(IV) oxides (32, 86). Random transposon mutagenesis selecting for non-Mn-oxidizing mutants in *P. putida* GB-1 produced mutants that were constitutive for siderophore production and did not produce MnO_2_ (40). In another study, PVD was found to complex Mn(III) more strongly than it binds Fe(III) (32). Consistent with the high thermodynamic favorability for Mn(III) complexation by PVD, PVD promotes the oxidation of Mn(II) to form PVD-Mn(III) (Fig. 1), even in Mn oxidation-deficient mutants (86). Only in the presence of bioavailable Fe, leading to the absence of PVD, do *P. putida* GB-1 and MnB1 produce MnO_2_. This is true whether PVD is produced by the cells or is exogenous (87). Furthermore, when Mn(III)-PVD forms, if Fe is made available to the cells, both GB-1 and MnB1 will oxidize the Mn(III) bound to the PVD, producing MnO_2_ and an altered PVD molecule. Interestingly, siderophores can also contribute to the reductive dissolution of Mn oxides (86, 88–90). Collectively, this work suggests that the fluorescent pseudomonads mediate the environmental chemistry of Mn even if they do not oxidize Mn(II) enzymatically.

### Mn oxidation in other pseudomonads

#### *P. putida* MnB1

*P. putida* MnB1, an isolate from a German water pipe biofilm (91), oxidizes Mn optimally at 24°C and neutral pH (92). In complex growth media, Mn oxidation is inhibited by high Mn(II) (360 µM) (92) and over 50 µM Fe(II) (93). High Fe(II) also decreases the expression of all multicopper oxidases (93), including ones with similarity to known Mn oxidases. Like *P. putida* GB-1, MnB1 forms reactive Mn oxides in biofilms (44, 94, 95).

While there is no genome available for this organism, some genes involved in Mn(II) oxidation by *P. putida* MnB1 have been identified by transposon mutagenesis (96). Caspi et al. identified involvement of genes in the c-type cytochrome maturation operon (genes *ccmE, F,* and *A*); transposon insertions in *ccmF* also disrupt Mn oxidation in *P. putida* GB-1 (40). Additional disrupted genes that impacted Mn oxidation in *P. putida* MnB1 included genes encoding the TCA cycle and carbon degradation (*sdhABCD, aceA, icd*) and tryptophan biosynthesis (*trpE*) (96). Interestingly, the *sdh* and *ace* mutants could be complemented by cross-streaking with WT strains or the addition of the central carbon metabolite malate. Tryptophan addition to the *trpE* mutant led to eventual Mn oxidation after several days. As tryptophan is a precursor in quinone biosynthesis, Caspi et al. suggested that this mutation may interfere with quinone production (96), and it was later shown (63) that this mutant could be complemented with pyrroloquinoline quinone (PQQ) *in vitro*, thereby suggesting that quinones may play a role in manganese oxidation. A sequenced genome of *P. putida* MnB1 could provide additional insight into the role these genes play in Mn oxidation in this and other *Pseudomonas* strains.

#### *P. putida* KT2440

Analysis of the genome of *P. putida* KT2440 reveals it contains the MnxS1S2R TCR, *xcmX*, *mnxG, mcoA,* and *mopA* (Fig. 5B). However, regulation of Mn(II) oxidation in *P. putida* KT2440 may be different from *P. putida* GB-1 given its reported requirement for low oxygen and its distinct siderophore (87, 97). In fact, when *P. putida* GB-1 is supplied with PVD_KT2440_ and Fe(III), both biofilm formation and Mn(II) oxidation were inhibited (87). Research to clarify what chemical conditions (e.g., O_2_, Mn, pH, Fe, etc.) allow for Mn oxidation in *P. putida* KT2440 would be valuable given its well-studied genetics, broad applications in environmental remediation, and its adoption as a synthetic biology chassis (98).

#### *P. resinovorans* and closely related strains

Several strains of *P. resinovorans* and related species have been isolated as Mn(II)-oxidizing bacteria from water treatment systems and sediment samples, including MOB-513, MOB-449, and MO-1 (10, 99–101). Strains MOB-513 and MO-1 (101) possess genes with moderate to high sequence similarity to the putative MCOs and heme peroxidases found in *P. putida* GB-1 (Table 1). The genome of the carbazole degrader *P. resinovorans* CA10 (NBRC 106,553) (102) also carries putative Mn oxidation genes (Fig. 5A) but, to our knowledge, its ability to oxidize Mn has not been studied.

In strain MOB 513, overexpression of a diguanylate cyclase to increase intracellular c-di-GMP levels was shown to increase both biofilm formation and Mn oxidation (100). Additionally, c-di-GMP increased expression of two of the three *mopA* homologs present in this strain but had no effect on expression of the putative Mn oxidase genes *mnxG and mcoA* or the third *mopA*. As described above, c-di-GMP also regulates FleQ activity (78), which was shown to play a role in the regulation of *mopA* oxidation in *P. putida* GB-1 (47). Thus, c-di-GMP and FleQ regulation of Mn oxidation may be widespread in the *Pseudomonas* genus.

In strain MOB-449, Mn oxidation appears to be related to the expression of cytochrome c terminal oxidases (99), suggesting a link to energy conservation. For example, qRT-PCR analysis revealed that expression of genes encoding aa3-type and cbb3-type cytochrome c oxidases is induced by Mn^2+^, while mass spectrometry showed upregulation of a cytochrome c1 (PetC), subunits of the cbb-type cytochrome c oxidase (CcoO1/CcoO2), and a protein involved in c-type cytochrome maturation (CcmE) in the presence of Mn^2+^. These proteins increase in abundance in the presence of Mn^2+^ at 18°C but not at 30°C, consistent with enhanced Mn oxidation at lower temperatures. As the genome is not available, whether this strain has any of the known putative Mn oxidases found in *P. putida* GB-1 and whether they have a role in Mn oxidation has not been reported.

#### *Pseudomonas* sp. DSV-1 and MS-1

*Pseudomonas* sp. DSV-1 and MS-1 were isolated from a compost pile in western Minnesota (12). The two strains are closely related to *P. psychrophila* (Fig. 5A) and can grow and oxidize Mn at temperatures as low as 4°C. Genome sequencing revealed that they encode proteins similar to MnxG and McoA, but not MopA. They also contain putative homologs to the MnxS1S2R TCR operon and the transport gene *xcmX* (Fig. 5A; Table 1). However, the contribution of these genes to Mn oxidation in DSV-1 and MS-1 is unknown. Random transposon mutagenesis selecting for non-Mn(II)-oxidizing phenotypes revealed that Mn oxidation in these strains is regulated by *rpoN*, which codes for the σ^54^ factor and implies a role for the σ^54^-dependent transcription factor MnxR similar to that seen in *P. putida* GB-1. Transposon insertions into genes that encode proteins in the TCA cycle and cytochrome c maturation also affected Mn oxidation. These results suggest broad similarities in regulation of Mn oxidation between *P. putida* strains DSV-1, MS-1, MnB1, and GB-1 and *Pseudomonas* MOB-449.

## POSSIBLE FUNCTIONS OF Mn OXIDATION IN PSEUDOMONADS

The presence of one or more Mn-oxidizing genes, the differing genome organization, and differences in Mn oxidation in response to the environment highlight the complexity of Mn oxidation in *pseudomonads*. In particular, the tight regulation of the oxidizing genes in response to environmental cues (e.g., Mn, oxygen, iron, temperature) suggests that Mn oxidation and biomineralization serve one or more functions that provide a competitive advantage to the individual cells involved in Mn oxidation, the broader population of cells within a species, or a community of diverse organisms. Despite the lack of published studies on function, several hypotheses can be considered, as described below.

### Metal homeostasis and protection from ROS

Mn(II) oxidation may have a role in Mn and Fe homeostasis and, by extension, oxidative stress protection (103, 104). Consistent with this proposed function, Mn(II) oxidation was linked to increased intracellular Mn concentration and increased protection from exogenous hydrogen peroxide stress in *P. putida* GB-1 (105). The formation of Mn oxides may thus provide a reservoir of solid-phase Mn that can consume ROS or can be tapped through Mn reduction when intracellular Mn(II) is needed. In addition to Mn homeostasis, Mn(II) oxidation may also play a role in or be affected by Fe homeostasis given the sensitivity of Mn oxidation to the extracellular concentration of aqueous Mn and Fe. Specifically, high concentrations of both Mn and Fe appear to inhibit Mn oxidation by *P. putida* MnB1, while low Fe, which promotes siderophore exudation, also disrupts Mn oxidation in MnB1 and GB-1. The relationship between Mn(II) oxidation, Mn and Fe homeostasis, and ROS protection should be investigated, especially considering that siderophores bind both Fe and Mn and therefore provide a direct link between Mn and Fe homeostasis.

### Mn oxidation as a possible mechanism for energy conservation

The oxidation of Mn to form Mn oxides could support energy conservation through multiple pathways. Mn(II) oxidation had long been proposed to serve as an energy source for bacteria. Recently, chemolithoautotrophic Mn oxidation was shown to occur in co-culture (82) (but not *Pseudomonas* species). In these co-cultures, cytochromes were hypothesized to be important for energy conservation (82). Subsequent enrichment cultures and genomic analysis of Mn(II)-oxidizing autotrophy from different environments provided additional evidence for the involvement of a cytochrome in chemolithoautotrophy(106) .

Although *Pseudomonas* species are not known to perform chemolithoautotrophy, they may be able to obtain maintenance energy in stationary phase from Mn oxidation, as suggested by transposon mutagenesis studies where mutations in genes involved in central metabolism (*sdhABC*, *aceA*), cytochrome *c* maturation (*ccmA, E, F*), and quinone synthesis (*trpE*) led to nonoxidizing phenotypes (12, 40, 96). Although the role of cytochromes and quinones in Mn oxidation has not been investigated in detail, the trafficking of electrons from Mn(II) to the electron transport chain or oxygen can be easily imagined given the known role of c-type cytochromes and quinones in electron transfer for energy conservation. But the exterior localization of Mn-oxidizing proteins makes their role in energy conservation less clear.

Recent work suggested that cytochromes may be required for Mn(II) oxidation in the biofilm-forming organism *Pseudomonas* strain MOB-449 (99). In this organism, in the presence of Mn(II) at low temperatures, the transcription of several cytochrome c terminal oxidase genes was induced and protein levels for PetC, a cytochrome c5, and CcmE, required for cytochrome c maturation, were increased. Based on these results, the authors suggested that the energy produced by Mn oxidation is conserved by cells at low temperatures. Similarly, two cold-tolerant Mn(II) oxidizers showed increased Mn(II) oxidation at 14°C versus 24°C (12). Whether Mn(II) oxidation provides *Pseudomonas* with additional conserved energy under conditions unfavorable for growth (e.g., cold, anoxic, nutrient-depleted) should be evaluated.

Mn(III,IV) species formed through Mn oxidation are known to function as electron acceptors under anoxic conditions (24, 25). This has not been shown with a Mn(II)-oxidizing *Pseudomonas* strain yet, but isolates related to *Pseudomonas fluorescens* have been shown to reduce Mn oxides (107). It is possible, then, that a Mn-oxidizing *Pseudomonas* species may be able to both oxidize and reduce Mn depending on the oxygen environment. Investigation of the pathways involved in biological Mn oxide reduction may provide insight into the potential for energy conservation in Mn oxidizing organisms. Additionally, clues about microbial Mn transformations may be obtained by analysis of the parallels with bacteria involved in Fe cycling (108).

Reduction of biogenic Mn oxides may also be coupled to the breakdown of complex organic carbon molecules into bioavailable carbon substrates. For example, pyruvate and other small-molecular-weight compounds form upon reaction of Mn oxides with complex natural organic matter (109, 110). Similarly, formate is known to form upon reaction of Mn oxides with carbohydrates (111). Biogenic oxides could thus mediate extracellular carbon oxidation to generate C1 (e.g., formate), C2 (e.g., acetate), or C3 (e.g., pyruvate) compounds that can serve as carbon and/or energy sources during respiration. Future research can shed light on the function of this trait in pseudomonads, including how the different Mn oxidases may serve to optimize energy conservation or carbon acquisition under different environmental conditions.

## THE PATH FORWARD

The *Pseudomonas* genus is fertile ground for studies on the distribution, evolution, and function of bacterial Mn oxidation. On one hand, the isolation and culturing of Mn-oxidizing pseudomonads have provided evidence for enzymatic Mn(II) oxidation in diverse environments, but complete biochemical characterization of the genes, regulatory pathways, and enzymes involved is lacking. On the other hand, many sequenced *Pseudomonas* genomes are publicly available, which allows for hypothesis testing not only regarding the mechanisms and regulation of Mn oxidation but also its evolution and ecology.

Figure 5 presents a phylogenetic survey of the distribution of potential Mn oxidizers in the *Pseudomonas* genus. Genes with similarity (expect value <1 × 10^−40^) to those shown to be involved unequivocally in Mn oxidation (i.e., *mnxG*, *mcoA*, *mopA*, *mnxS1*, *mnxS2*, *mnxR,* and *xcmX*) are present in about one-third of 52 examined *Pseudomonas* genomes (Fig. 5A). Pseudomonads with putative Mn oxidation genes are found in several clades within the genus, including the Resinovorans, Aeruginosa, Putida, and Fluorescens clades (Fig. 5A). This may reflect the polyphyletic nature of the *Pseudomonas* genus (112), with the different clades evolving from separate common ancestors. However, not all members within a clade possess Mn oxidation genes. For example, *P. aeruginosa* apparently lacks Mn oxidation genes and has not been reported to oxidize Mn, while *P. knackmussi* in the same clade has Mn oxidation genes, although its ability to oxidize Mn is unknown. A more granular analysis shows that among 34 complete *P. putida* genomes on the IMG/M site, only two lacked any matches to the Mn oxidation genes (*P. putida* NX-1 and PC2), while the rest carried at least three of the five genes (Fig. 5B). Additionally, 20 out of 34 strains shown in Fig. 5B possess all three Mn oxidase genes reported to date for *pseudomonads* (*mnxG*, *mcoA*, *mopA*). Still, these *P. putida* strains are quite diverse. When compared using Average Nucleotide Identity values (% ANI), many of these strains, including GB-1, could be considered separate species from *P. putida* (less than 95% ANI, K. Geszvain, unpublished).

In Fig. 6, we show the putative operons that encode *mnxG*, *mcoA,* and the MnxS1S2R TCR. The putative *mnxG* operon contains six genes, including *mnxG,* each of which is present in *P. putida* GB-1, *Pseudomonas* sp. MS-1, and *P. resinovorans* MO-1 (12, 113). The putative *mcoA* operon contains five genes in *P. putida* GB-1, but only two of these genes, *mcoA* and the gene located immediately downstream (a predicted SCO1/SenC protein), are conserved in *Pseudomonas* MS-1 and *P. resinovorans* MO-1. Organization of the MnxS1S2R TCR genes across these organinisms is more variable than that of *mnxG* and *mcoA*. In *Pseudomonas* sp. MS-1, the *mcoA* gene and the SCO1/SenC gene are found between *mnxS1* and *mnxS2,* combining the two putative operons into one. In *P. resinovorans* MO-1, the genome has two *mnxS2*/*mnxR* operons but lacks *mnxS1*. Interestingly, the SCO1/SenC genes in the *mnxG* and *mcoA* putative operons (12) may function as copper chaperones for MnxG and McoA. Future research can examine whether these patterns in gene organization reflect different regulatory strategies or even different functions for Mn oxidation.

The presence of up to three Mn oxidases in pseudomonads (Fig. 5 and 6) raises many questions. Variable responses to growth conditions such as media type, Mn concentration, and oxygen availability (48, 51, 59), combined with cell-to-cell variation within a population, suggest that the different Mn oxidases are specialized and regulated for different environmental conditions. By merging mutagenesis and imaging approaches using reporter strains to untangle the regulation of Mn(II) oxidation in *Pseudomonas,* clues regarding the evolutionary origin and function of Mn(II) oxidation will likely emerge. This knowledge will have large implications for Earth and environmental science as well as in fundamental microbiology.

Mn oxidation leads to the precipitation of Mn oxides on or near the cell’s surface (Fig. 2). How do Mn-oxidizing bacteria protect themselves from the potentially deleterious effects of these potent oxidants? Mn biomineralization coincides with the production of extracellular polymeric substances (EPS, Fig. 2A) (44, 79). High-pressure freeze-substitution preparation of laboratory cultures shows that Mn oxides are near but not directly bound to the cell envelope (Fig. 2B and C). Thus, the biofilm matrix itself may provide bacterial cells with both physical and chemical protection against the high reduction potential of the Mn oxides (114). Studies on the role of EPS should consider that Mn oxidation is regulated by pathways involved in biofilm production (e.g., c-di-GMP in *P. resinovorans* or FleQ in *P. putida*). The role played by the biofilm matrix in modulating the reactivity and proximity of biogenic Mn oxides to the bacteria, and subsequently their ability to retrieve an advantage from the proximity of these minerals, thus requires further investigation.

Manganese-oxidizing bacteria, and in particular, Mn-oxidizing *pseudomonads,* also have great potential for biotechnological applications (14, 115–117). Broad application, however, requires further knowledge about the biochemistry of the enzymes catalyzing Mn oxide biomineralization. For example, the enzymes could be engineered to produce new biomaterials with specific chemistries and stability under conditions relevant for bioremediation of metal pollution, degradation of organic contaminants, recovery of critical minerals or for the production of nanomaterials that possess novel electronic, optical, chemical, and catalytic properties. The morphology and surface chemistry of biogenic Mn oxides can be varied by changing the protein-to-substrate ratio or the Ca^2+^ concentration (118). The possibilities are vast.

To effectively harness these opportunities, a deep understanding of the mechanism of Mn oxide mineral formation is needed. How do these different proteins take soluble Mn^2+^ and make a MnO_2_ particle? Do the properties of the enzymes dictate the properties of the biogenic Mn oxides? How can the different enzyme systems be controlled? We have gained some insights to these questions through studies of the *Bacillus* sp. Mnx system (53), but similar progress must be made in *Pseudomonas*. That *Pseudomonas* spp. are genetically tractable, easily culturable, and offer a plethora of Mn oxidases should greatly motivate biochemical studies of their Mn oxidases.
